# No trauma-related diagnosis in emergency trauma room whole-body computer tomography of patients with inconspicuous primary survey

**DOI:** 10.1007/s00068-024-02511-0

**Published:** 2024-04-18

**Authors:** Arnold J. Suda, Julia Pepke, Udo Obertacke, Holger Stadthalter

**Affiliations:** 1 AUVA Trauma Center Salzburg, Department for Orthopaedics and Trauma Surgery, Dr-Franz-Rehrl-Platz 5, 5010 Salzburg, Austria; 2 University Medical Centre Mannheim, Medical Faculty Mannheim of Heidelberg University, Centre for Orthopaedics and Trauma Surgery, Theodor-Kutzer-Ufer 1-3, 67168 Mannheim, Germany

**Keywords:** Whole-body CT, Primary survey, ATLS®, Trauma mechanism

## Abstract

**Purpose:**

Whole-body computer tomographic examinations (WBCT) are essential in diagnosing the severely injured. The structured clinical evaluation in the emergency trauma room, according to ATLS® and guidelines, helps to indicate the correct radiological imaging to avoid overtriage and undertriage. This retrospective, single-center study aimed to evaluate the value of WBCT in patients with an inconspicuous primary survey and whether there is any evidence for this investigation in this group of patients.

**Methods:**

This retrospective, single-center study was conducted with patients admitted to a maximum-care hospital and supraregional trauma center in Germany and part of the TraumaNetwork DGU® in southwest Germany between January 2012 and November 2017. Hospital files were used for evaluation, and WBCT was carried out using a 32-row MSCT device from Siemens Healthineers, Volume Zoom, Erlangen, Germany. For evaluation, non-parametric procedures such as the chi-square test, *U* test, Fisher test, and Wilcoxon rank sum test were used to test for significance (*p* < 0.05).

**Results:**

From 3976 patients treated with WBCT, 120 patients (3.02%) showed an inconspicuous primary survey. This examination did not reveal any trauma sequelae in any of this group. Additionally, 198 patients (4.98%) showed minor clinical symptoms in the primary survey, but no morphological trauma sequence could be diagnosed in WBCT diagnostics. Three hundred forty-two patients were not admitted as inpatients after WBCT and discharged to further outpatient treatment because there were no objectifiable reasons for inpatient treatment. Four hundred fifteen patients did not receive WBCT for, e.g., isolated extremity trauma, child, pregnancy, or death.

**Conclusion:**

Not one of the clinically asymptomatic patients had an imageable injury after WBCT diagnostics in this study. WBCT should only be performed in severely injured patients after clinical assessment regardless of “trauma mechanism.” According to guidelines and ATLS®, the clinical examination seems to be a safe and reliable method for reasonable and responsible decision-making regarding the realization of WBCT with all well-known risk factors.

## Introduction

In the acute care of seriously injured patients, stabilizing vital functions is a top priority. It is, therefore, particularly important to enable and carry out the necessary medical measures as quickly as possible. In order to optimize this special care, the German Society for Trauma Surgery (DGU) has defined the necessary local conditions and diagnostic procedures in the “White Paper on Serious Injury Care” in recent years [1] and thus defined standards of care and quality in the emergency trauma room (ER). These apply in Germany and some neighboring countries (Switzerland, Austria, Luxembourg, Belgium). Standardized protocols and algorithms, specially defined for this complex management, are regularly trained in the hospitals participating in the DGU’s certified trauma centers. Within the framework of diagnostics carried out according to established procedural standards, whole-body computer tomographic examinations (WBCT) are primarily used in hospitals worldwide that care for seriously injured patients for the reliable detection of potentially life-threatening injuries after an accident. WBCT is done in support of the equally standardized clinical examination and imaging sonographic and radiographic diagnostics according to ATLS® [2]. The regular use of WBCT according to the S3 guideline valid in Germany is, however, only indicated for severely injured but stable patients for several reasons [3]. Often, upon arrival in the emergency department of patients who have been pre-hospital classified as potentially severely injured according to the trauma mechanism, after the initial clinical examination/primary survey according to ATLS® [2], the impression is that they might not be as seriously injured as initially assumed. Nevertheless, polytraumatized patients have considerable economic consequences in Germany, which is highly competent in emergency medicine [4–6]. Diagnosis and life-saving treatment should be applied immediately [7–9]. Underestimation or overestimation of injury severity (“overtriage” vs. “undertriage”) cannot always be avoided in the pre-hospital phase [10–13]. However, the best possible assessment can be achieved through appropriate training (e.g., ATLS® courses) and, in addition, from the experience of the attending emergency personnel [14–17]. Because of the high risk of faulty communication due to the stressful situation and time pressure, several schemes have been created as communication aids for this critical moment of transfer from pre-hospital to clinical further treatment [18]. The ATLS® training concept aims to provide standardized, time-optimized, and problem-oriented diagnostics and acute treatment from when the injured patient arrives at the treating hospital, including focused abdominal sonography of trauma patients (eFAST) [19–24]. Multislice whole-body CT (MBCT) of the patient is carried out immediately after the primary survey following the workflow specifications. If necessary, the polytraumatized person must first be cardiopulmonary stabilized. Today, computed tomography is a very effective and time-saving method of completing diagnoses and documenting the course of diseases in images [25]. Since 2004, it has also played an essential role in caring for seriously injured patients within scientifically established procedures. The Federal Office for Radiation Protection recommends 1 mSv per year as the maximum effective dose for ordinary citizens to minimize stochastic (e.g., cancer) and deterministic (direct skin damage) risks [26]. The maximum permissible exposure to humans in the individual and occupational sectors with increased radiation exposure (e.g., medical professions, flight personnel) and pregnant women is specified. Medical examinations are not subject to any limits set by the Radiation Protection Act. Computer tomography works based on X-rays—the examination procedure of a whole-body CT with a radiation dose of approximately 10–20 mSv per examination, in addition to intravenous contrast medium administration, therefore, represents acute stress for the patient’s body in the sense of X-ray exposure and strain on the kidney function. Contrast medium allergies also occasionally occur clinically in connection with the examination. Malignant diseases diagnosed retrospectively as a result of irreversible DNA damage can be challenging to speak of independently of such radiation exposure that has taken place. The standardization of a whole-body CT examination, accompanied by a high dose of ionizing radiation to the patient, is therefore questionable in principle due to the incalculable risk of potential side effects, even in the long term. This retrospective, single-center study aimed to examine trauma diagnosis in WBCT of patients with an inconspicuous primary survey, and the question if this still justifies whole-body CT diagnostics in ER in cases with a severe trauma mechanism.

## Methods

In this retrospective monocentric study, all patient files of those that were treated in the trauma ER of the University Medical Centre from January 2012 to November 2017 were included in the survey. The University Medical Centre Mannheim (UMM) is a maximum-care hospital, a supraregional trauma center in Germany, and part of the TraumaNetwork DGU® in southwest Germany. Raw data for the analysis are CT data, radiological findings, clinical ER protocols, physician and discharge reports, and laboratory chemistry tests from hospital files. A statistical evaluation of the injury severity concerning the accident mechanism and CT-related findings was performed. Data was collected in the de-identified form via the daily work protocols of the UMM over the period as mentioned above, the SAP iMED software established on site, and the local intranet laboratory value server. There was potential sampling and selection bias as this was a single-center study in a mid-west German hospital and a potential incidence‐prevalence bias. The parameters listed in the context of the file inspection were finally statistically evaluated to record an incidence of trauma consequences in the diagnostics carried out. The computed tomography scanner used for imaging in the context of ER protocols during the evaluated studies was a 32-row MSCT device from Siemens Healthineers, Volume Zoom, Erlangen, Germany. Patients were positioned with their arms raised in the “head first” position. The monitoring of the vital parameters, if necessary, under invasive or non-invasive ventilation, is continued throughout the entire examination to check and guarantee the cardiopulmonary stability of the patients. The WBCT includes a contrast medium examination (CT) with the sequencing of the neurocranium, in some cases also of the paranasal sinuses and orbits in the case of midface injuries, with the cervical spine (C-spine) and thorax to pelvis with soft tissue, bone, and thoracic lung windows in order to have a rapid and reliable overview of any potentially life-threatening injuries that may be present [27, 28]. The secondary reconstruction of the sagittal bone window is calculated as standard, and in the case of fractures, the coronary layers are also calculated. If necessary, sagittal and coronary layers of the orbit and cervical spine also exist. The contrast medium (KM) was administered bolus-triggered with 120 mL iodine-containing contrast medium, in this case, Ultravist 300®, Schering, Berlin, Germany, in the early arterial phase of the thoracic examination. Thirty seconds after KM administration, in the portal venous phase, the abdomen is examined to detect intra-abdominal lesions of the parenchymatous organs. Alternatively, a contrast medium containing gadolinium is used, for example, in cases of known iodine allergy. The advantage is better toleration and thus safer use than contrast media containing iodine. The direct review of the recorded images is carried out by a medical radiologist at the diagnostic monitor. A verbal interdisciplinary handover to the treating departments follows this. Subsequently, a decision is made on the further treatment method. Within a maximum of 1 h, the respective radiologists will send a preliminary written report to the team. Indications for ER treatment, including WBCT, were according to German S3 guidelines for polytrauma treatment. The Abbreviated Injury Scale (AIS) was used for standardized assessment of the degree of injury [29]. The AIS has been continuously developed over the years [30–32]. Misjudgments of injury severity are a possible source of error, which can have consequences for the further care of the injured person [33, 34]. In this study, a modified AIS code was applied for better comparability in the sense of a subdivision of the individual injury organs equivalent to the division of the examined body regions in the CT examination. The ISS is currently the most widely used score worldwide for assessing the lethality risk of acute trauma injuries. The Injury Severity Score (ISS) is calculated from a specific combination of injuries of the traumatized person, namely as the sum of the squares of the three highest AIS codes (points of the three most severely injured body regions) [29, 35–39]. The study was conducted following the Declaration of Helsinki as amended. The ethical approval of the Ethics Committee of the Medical Faculty Mannheim of the University of Heidelberg of 17.10.2017 bears the mark 2017-844R-MA and certifies no ethical or professional concerns and no need for informed consent. The requirements were fulfilled through a purely retrospective data evaluation without further examinations, interviews, or patient contact. No additional investigations or determinations were made. The data was analyzed anonymously following the provisions of the Federal Data Protection Act. The medical confidentiality of data processing was and will continue to be observed. Advice and support were obtained from the Department of Medical Statistics, Biomathematics, and Information Processing at the Mannheim Medical Faculty of Heidelberg University. The following software was used to analyze the data: SAS 9.4 (SAS Institute Inc., SAS Campus Drive, Cary, NC 27513, USA). For evaluation, descriptive statistics and non-parametric procedures such as the *U* test, Fisher–Yates test, and Wilcoxon rank sum test were used to test for significance (*p* < 0.05).

## Results

**Fig. 1:**
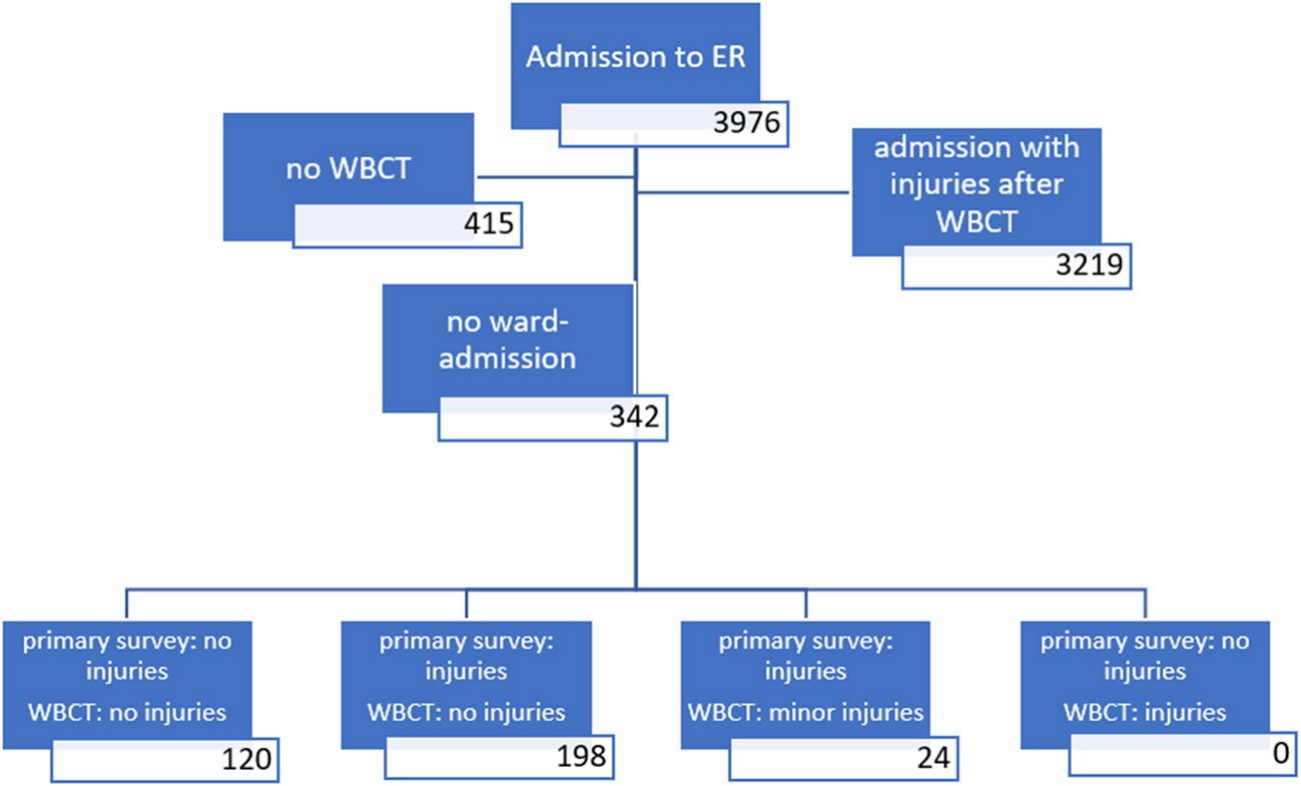
Flowchart of results

**Fig. 2:**
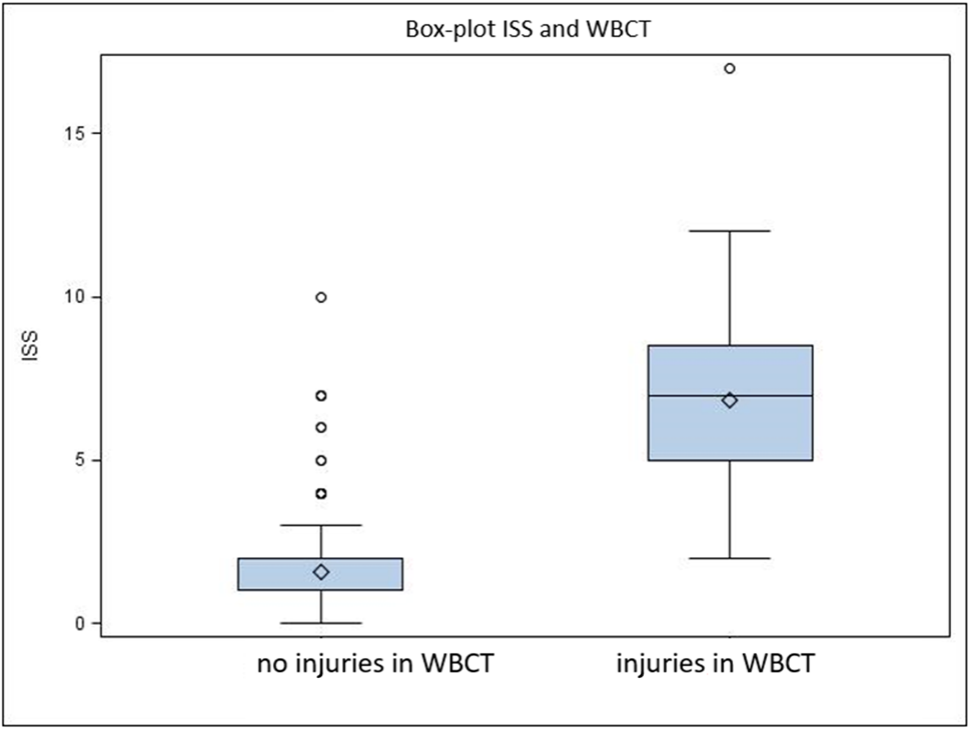
New boxplot of ISS to WBCT injuries detected

From January 2012 to November 2017, 3976 patients were treated by the orthopedic trauma surgery center in the ER of Mannheim University Medical Centre, a level-I trauma center in mid-west Germany. We found 342 patients from this total collective, 241 men and 101 women (mean age 39.7 ± 15.9 years, range 15 to 85 years), who were not admitted as inpatients after WBCT and discharged to further outpatient treatment because there were no objectifiable reasons for inpatient treatment. However, no morphological trauma sequence could be diagnosed in WBCT diagnostics: the 24 patients (7% of these 342 patients) who actually showed minor injury in WBCT all showed clinical signs of (minor) injuries as well (*p* < 0.001). One hundred twenty patients (3.02% of all patients) were utterly inconspicuous in the primary survey who nevertheless underwent WBCT diagnostics due to trauma mechanisms. This examination did not reveal any trauma sequelae in any of this group. In the primary survey, 198 patients (4.98%) showed minor clinical symptoms such as pain or superficial soft tissue injuries but no injury in WBCT (Fig. 1). The “number needed to scan” to diagnose an injury on CT among the entire patient population was 1:19, which is also among the population of road traffic injuries in this study. For various reasons, 415 patients did not receive WBCT (e.g., isolated extremity trauma, child, pregnancy, death). By far, the most frequent trauma was a motor vehicle crash (MVA) in approximately 87% of the patients who were admitted to the shock room diagnosis. The second most frequent cause was falls from different heights (8%), followed by physical violence (4%). Of the MVA patients, 76% had been involved in a car crash—of which 50% were frontal, 28% were side-impact, 11% were rollover, and the rest were either from another direction or unknown. Of the cases in the passenger car collective, 68% were male, and 32% were female. 12% had crashes with a motorized two-wheeler (motorcycle/scooter). In addition, 7% were involved in crashes as cyclists and 2% lorry drivers; 1% were involved in traffic crashes with other types of motor vehicles (quad bikes and trains). From the group of traffic crashes, 2% were also injured as pedestrians in road traffic. Most common injuries suspected in primary survey were head injuries, thoracic injuries, pelvic and abdominal injuries, spine, and extremities with no differences between patients with or without injuries found in WBCT (*p* > 0.05 using *U* test and Wilcoxon rank sum test). It was clear that ISS was significantly lower using the Fisher–Yates test in patients without injuries detected in WBCT as shown in Fig. 2 (*p* < 0.001).

## Discussion

Not one of the 120 clinically asymptomatic patients had an imageable injury after WBCT diagnostics in this study. The results suggest that WBCT should only be performed in patients with suspected severe injuries after clinical examination regardless of “trauma mechanism” due to associated exposure to X-rays and all their possible consequences.

This study has several limitations: First, the study design was retrospective and single-center, which could have affected the results. With a multicenter study with international partners, results could differ due to different diagnostic and treatment protocols and larger sample size. Second, the indication for treatment in the ER was set according to German S3 guidelines, which may lead to different indications in different countries. Third, the assessment was done using the standardized ATLS® algorithm and the primary survey. Using a different method, the results of clinical examinations may differ. Previous studies have already questioned the indication for standard WBCT in polytrauma diagnostics. The advantages of WBCT diagnostics with gadolinium-containing contrast media are clear: it is usually available in the vicinity of the hospital’s shock room and thus quickly, and it is also a welcome modern procedure for rapid and reliable diagnosis and therapy planning. Huber-Wagner et al. have already shown in several retrospective multicenter studies the significant advantage of using a rapid WBCT examination in severely injured and polytraumatized (ISS ≥ 16) patients in terms of a measurably reduced mortality and, therefore, higher survival probability when using this examination method, which speaks for an increase in diagnostic safety [40]. This also applies to hemodynamically unstable patients [40, 41]. According to these studies, the “number needed to scan” to save a life by this diagnostic measure is potentially 53 in hemodynamically stable and 25 in unstable patients. In a comparison of two patient collectives treated in a level-1 trauma center in 2001–2003 (group 1—conventional emergency diagnostics including FAST) and 2004–2006 (group 2—introduction of whole-body CT protocol), Wurmb et al. also found a shorter time from diagnostics to emergency surgery in the group that had undergone whole-body CT diagnostics. However, according to the authors, the patients in this group were also more severely injured in comparison. Therefore, the authors assume an improved outcome in terms of mortality due to MSCT diagnostics. It is under discussion whether a targeted organ-related CT diagnosis would be sufficient to confirm the diagnosis or whether this is indicated at all in the case of a suspected but regionally limited physical injury within the scope of the primary survey, in the case of an AIS < 3 or the case of absent signs of injury. Among others, Sierink et al. published the results of the prospective REACT-2 study in 2016. They could not show any advantages in terms of mortality during hospitalization by performing immediate WBCT diagnostics. However, they showed a clear advantage in terms of time compared to conventional diagnostics with essential imaging with trauma sonography and X-ray after clinical examination in less severely injured patients [42]. CT imaging again showed a clear diagnostic and temporal advantage in severely injured patients in this study. The group with conventional diagnostics with selective CT examination was exposed to a significantly lower radiation dose. The prognosis of the outcome of acutely injured trauma patients receiving initial care in trauma centers has been investigated in a publication by the working group of Nolte et al. from 2020 [43]. Over a period of 9 years, more than 64,000 patients with a RISK II score of < 10% were examined with regard to the occurrence of sudden death in the course of hospitalization. Patients with ISS < 4 (RISC cannot be applied in this case) and patients with low-grade injuries (maximum AIS ≤ 2) were excluded. The overall hospital mortality rate among the patients included in the study was 2.1%, of which 22% died within the first 24 h. They found that the highest risk of dying within 24 h was in patients with severe chest or abdominal trauma (each AIS ≥ 3) and systolic blood pressure ≤ 90 mmHg. Patients over 60 years of age were most likely to die if they were hospitalized for longer than 24 h. They declared the presence of these characteristics as “red flags” that should be taken into account in order not to underestimate presumably less injured patients. Patients ≥ 65 years, in particular, often have a history of previous illness and home medication at the time of the accident, which may also include anticoagulants. In the event of severe trauma, this combination, even with a lesser extent of injury, brings with it an increased mortality rate, as Kirshenbom et al. also published in a study in 2017 [44]. Several studies have already been conducted on the outcome of patients with comorbidities at a higher age [45–47]. However, most of these are not satisfactorily conclusive or sometimes come to controversial results. In the meantime, several other working groups are currently dealing with similar questions. Recently, Reitano et al. published studies that questioned the general indication of a whole-body CT examination in apparently minor injured patients with corresponding inconspicuous clinical, sonographic, and laboratory-chemical indications [48, 49]. They referred to the known current ATLS® criteria [2], which, when applied conscientiously, gave a very concrete indication of the severity of the patient’s injury. They advised taking these first and deciding for or against CT diagnostics accordingly. In 2015, Davies established a scoring system to create a more unambiguous objective indication for WBCT examination after trauma, the “Manchester Trauma Imaging Score” (ManTIS) [50]. Two hundred fifty-five patients were included, of whom 16% had polytrauma, 42% had only minor injuries, and another 42% had no injuries. In the 42% with no clinical evidence of relevant injury, the absence of relevant injury severity was confirmed on CT (except for one person examined). The sensitivity of their system is reported to be 97%, and the specificity is 56%. They state that the probability of survival for AIS of 1 or 2 as singular values is 99.4% and 99.3%. Above an AIS of 3 as a singular injury, mortality increases significantly. Therefore, if there is any doubt about the presence of relevant injuries, they recommend, after a thorough examination of the patients, to refrain from a WBCT to benefit the patients and to reduce the radiation dose. Like Reitano et al., they ultimately appealed to trust in the thorough examination results of the patients and encouraged them to forego the WBCT examination on a case-specific basis and to choose focused imaging if necessary. Some concrete disadvantages and risks of an MSCT examination are now known. For example, ionizing radiation is known to predispose to malignant diseases. In addition, the risks of contrast medium administration must be mentioned, such as allergic reaction, nephrogenic systemic fibrosis with accompanying joint stiffness, and also damage to internal organs, such as acute liver but not kidney failure in patients who usually have corresponding previous illnesses [51]. However, it can also affect patients without known previous illnesses. Particularly in pregnancies, the indication for the administration of contrast media and, of course, also for radiation-intensive diagnostics must be strictly defined. According to the DGU® Annual Report 2021, 92,484 patients with injuries with at least AIS > 2 were examined in the trauma network in 2018–2020 as a “basic collective.” Due to the high number of less injured patients and subsequent increased imprecision of the results, the base collective was defined as “all patients with a maximum AIS severity (MAIS) ≥ 3 or MAIS 2 who either died or were in intensive care.” Most relevant injuries in the baseline collective were diagnosed in the head/skull (45.9%) and thorax (45.4%), followed by spinal injuries (29.4%), extremity injuries (arms 29.2%, legs 23.2%), and pelvic (15.4%) and abdominal injuries (14.1%). With a few exceptions, the study conducted here dealt with patients from the non-basic collective of the DGU® annual report, i.e., those with a maximum AIS of 2 survived and did not require treatment in intensive care. The injury pattern in this group was also distributed proportionally among the body regions, as mentioned above, analogous to the more seriously injured patients of the DGU® [52]. Assessing the severity of an injury to the patient and avoiding “overtriage” for various reasons are significant challenges for every medical professional. This is strongly dependent on the professional experience of the doctors, as well as on individual self-confidence, familiarity, and routine in standardized work processes. The decisions may mean that the patients are exposed to a not insignificant amount of radiation for diagnostic purposes, saving time and leading to a perceived higher level of diagnostic certainty. However, this comes at the price of an unknown increase in the probability of developing a malignant disease in the long term due to radiation exposure and, last but not least, possible undesirable side effects of the applied contrast medium. Individual case analyses show, however, that there is indeed a specific mortality rate, even in the case of injury severities assessed more quickly in the primary survey after trauma triggering the shock room procedure. On the contrary, the injury severity is sometimes significantly lower than the investigator initially assumed. The annual report of the German Society for Trauma Surgery of 2019 describes 5305 patients with a maximum AIS severity level of 1 for 2018; of these, 33 (0.6%) patients died [52]. A comparable rate was also found in the previous year’s DGU® reports. This should be kept in mind when assessing the morbidity and mortality risk of patients arriving in the emergency department after trauma triggering the shock room procedure when planning the diagnostic procedure within the framework of the primary survey.

## Data Availability

No datasets were generated or analyzed during the current study.
